# Seasonal Cyclicity in Trace Elements and Stable Isotopes of Modern Horse Enamel

**DOI:** 10.1371/journal.pone.0166678

**Published:** 2016-11-22

**Authors:** Niels J. de Winter, Christophe Snoeck, Philippe Claeys

**Affiliations:** Department of Analytical-, Environmental-, and Geochemistry, Vrije Universiteit Brussel, Pleinlaan 2, B-1050, Brussels, Belgium; Max Planck Institute for Evolutionary Anthropology, GERMANY

## Abstract

The study of stable isotopes in fossil bioapatite has yielded useful results and has shown that bioapatites are able to faithfully record paleo-environmental and paleo-climatic parameters from archeological to geological timescales. In an effort to establish new proxies for the study of bioapatites, intra-tooth records of enamel carbonate stable isotope ratios from a modern horse are compared with trace element profiles measured using laboratory micro X-Ray Fluorescence scanning. Using known patterns of tooth eruption and the relationship between stable oxygen isotopes and local temperature seasonality, an age model is constructed that links records from six cheek upper right teeth from the second premolar to the third molar. When plotted on this age model, the trace element ratios from horse tooth enamel show a seasonal pattern with a small shift in phase compared to stable oxygen isotope ratios. While stable oxygen and carbon isotopes in tooth enamel are forced respectively by the state of the hydrological cycle and the animal’s diet, we argue that the seasonal signal in trace elements reflects seasonal changes in dust intake and diet of the animal. The latter explanation is in agreement with seasonal changes observed in carbon isotopes of the same teeth. This external forcing of trace element composition in mammal tooth enamel implies that trace element ratios may be used as proxies for seasonal changes in paleo-environment and paleo-diet.

## 1. Introduction

Records from fossil tooth bioapatite have often been used to reconstruct paleo-diet and paleo-environment (e.g. [1] [2] [3]; [4] [5] [6]). Because of the resistance of enamel bioapatite to geochemical alterations after burial, stable oxygen and carbon isotope ratios in this biomineral are featured in various studies (e.g. [7] [8] [9] [10]). Mammal tooth enamel has proven to be an ideal recorder of paleo-seasonality thanks to this high resistance to diagenesis and its incremental growth, allowing the recovery of high temporal resolution records ([11[12] [13]). In addition, geochemical proxy records of teeth from humans and other mammals have been used in a range of archaeological studies to answer questions about past diet, cooking practice, mobility and environmental change ([2] [14] [15] [16] [17] [18]). An added advantage of using mammal teeth for sub-annual environmental reconstructions is the possibility of combining multiple teeth of the same individual to create a composite time series. This combination allows the construction of longer continuous records of seasonal variations in paleo-environments during the years in which the teeth mineralized ([2] [19] [20]). In this study, a tooth row from a modern horse is used as environmental recorder to investigate the robustness of new paleo-seasonality proxies in tooth enamel that can be applied to fossil samples. More specifically, conventional stable isotope analysis is combined with μXRF analysis on the same samples to study the changes in trace element concentrations in horse enamel over seasonal time scales. The use of trace element concentrations in tooth enamel as proxies for paleo-environmental and paleo-dietary conditions of the animal during tooth formation is evaluated.

The practice of paleo-seasonality reconstruction based on the analysis of stable carbon and oxygen isotopes is established in various common domestic mammal taxa (e.g. cattle: [21], pig: [20], sheep: [11] [22] and horse: [13] [23]. However, the use of trace element analysis for this same purpose remains largely unexplored. While combining trace element records and stable isotope profiles is common practice in paleo-seasonality reconstructions from other, often invertebrate, paleo-seasonality archives, such as bivalves and corals (e.g. [24] [25] [26] [27] and references therein), the use of such a multiproxy approach in the study of enamel records remains rare ([10] [28]). Various new methods of fast, high-resolution and non-destructive elemental analyses are now widely available (e.g. [29] [30] [31]), enabling the measurement of trace elements in fossil material without altering the samples. The application of trace element proxies for paleo-environmental reconstructions from tooth enamel using these new methods opens up a whole range of opportunities to reconstruct paleo-seasonality, most noteworthy in terrestrial ecosystems where data for seasonality reconstruction is sparse. In order to investigate the use of trace element analyses as a proxy for paleo-seasonality, results of trace element concentrations analyzed using a novel laboratory micro X-Ray Fluorescence (μXRF) scanning method are combined with conventional stable isotope analysis on the carbonate fraction of horse enamel in an effort to study the expression of seasonality in trace elements. In addition, this study reports the first use of the μXRF line scanning method for non-destructive, high-resolution measurements of trace element abundances in mammal teeth and discusses the reliability of this method for quantitative trace element analysis in bioapatite.

## 2. Background

### 2.1 Modern horse dentition

Modern horses (*Equus caballus*) have a hypsodont dentition ([32][33]) causing them to grow high-crowned cheek teeth. Horse molars and premolars grow to about 8–9 cm length and their relatively fast growth rate of 3–4 cm/yr ([34]) allows the construction of multi-year geochemical records with a high (monthly) temporal resolution. The evolution of equids is well-studied, and modern horses form the extant species in a long lineage of fossil equid ancestors ([35] [36]). The nearest living relative principle justifies the use of modern horse as an analogue for its ancestors and allows the extrapolation of proxy relationships established on modern horses for the interpretation of fossil equid tooth records (e.g. [37] [38] [39]). This makes horses an interesting modern analogue taxon potentially enabling terrestrial paleo-climate reconstruction from the Early Eocene up to modern times ([13] [37] [38] [40] [41]).

The eruption and mineralization sequence of modern horses varies between races, but the timing of mineralization of horse cheek teeth is known within a 1 to 3 months range ([34]). This knowledge allows construction of a composite time series from multiple individual teeth. The eruption sequence of modern horses is described in [42] and [43] and the mineralization scheme was more recently studied by [34]. The first molar (M^1^), the first permanent tooth to be formed, erupts around month 11. It is followed by M^2^ around month 23, the second premolar (P^2^) appears around month 30 and P^3^ around month 34. Finally, P^4^ (eruption around month 46) and M^3^ (erupting around month 48) mineralize almost simultaneously. Mineralization of enamel is known to continue after eruption and after the maximum length of the tooth is reached, especially in molars ([34]). The total sequence of teeth covers a timespan of over 4 years ([34] [38]).

### 2.2 Bioapatite in mammal tooth enamel

Mammal tooth enamel is composed of an inorganic mineral fraction of crystalline bioapatite, with organic matter and water. The chemistry of the bioapatite is well studied and its composition can be approximated by the chemical formula: (Ca, Na, Mg, Ba, Fe, Sr, Zn, []) _10_(PO_4_, HPO_4_, CO_3_)_6_(OH, F, Cl, CO_3_, O, H_2_O, []) _2_ ([44] [45] [46] [47]) where [] represent potential vacancies in the crystalline structure. This formula shows that there are three phases in the bioapatite structure: cations (mostly Ca), phosphate, and the so-called channel-filling ions (e.g. OH). In enamel bioapatite, phosphate and the channel-filling ions are partly replaced by carbonate (CO_3_) ([45] [46] [48]). A range of trace element cations can substitute for Ca in the bioapatite structure, while anions like F^-^ and Cl^-^ are present in traces in the channels between the calcium and phosphate groups, replacing the hydroxyl-groups. Besides these groups, there is also room in the hydration layers between bioapatite crystals in enamel where water, organic matter and carbonates can be found ([49]). The presence of carbonates and organic matter in and between the bioapatite crystals causes defects in the hexagonal structure of bioapatite, which makes the more substituted bioapatites (such as bone and tooth dentine) prone to degradation and less stable in the archeological and fossil record compared to enamel bioapatite ([49] [50]).

### 2.3 Isotopes in bioapatite

Oxygen isotope ratios in the carbonate fraction of tooth bioapatite (δ^18^O_c_) are shown to be related to the oxygen isotope composition of the animal’s body fluid, which in large mammals is approximately 2–3‰ more enriched than the isotope composition of ingested water ([19] [22] [51] [52] [53] [54] [55]), which is driven by local precipitation and evaporation ([51] [56]). In coastal Northern Europe, the oxygen isotope composition of surface water is ^18^O-depleted (δ^18^O ≈ -10 ‰) in winter and enriched in ^18^O (δ^18^O ≈ -6 ‰) in summer ([57] [58] [59]; Kevin De Bondt (VUB), personal communication). This seasonal signal is reflected in δ^18^O_c_ of horse tooth enamel and enables the reconstruction of paleo-seasonality. For juvenile mammals, the δ^18^O_c_ values of teeth mineralized during the weaning period can be higher, because the water ingested through milk is enriched in ^18^O with respect to the drinking water of the mother ([54] [60]). In case of modern feral horses and zebras, which wean their young within the first 8–9 months after birth ([61]), the only permanent teeth mineralizing during the weaning period are the first molars ([34] [54]). As a consequence of weaning, only a part of the first molar of horses that mineralizes during weaning may show elevated δ^18^O_c_ values.

Carbon isotope composition (δ^13^C_ap_) in mammal teeth are related to the diet of the animal ([62] [63]). They have yielded good results in studies of long-term variation in floral ecosystems, such as changes between dominance of C_3_ and C_4_ vegetation in diet ([8] [64] [65] [66]). In studies of (intra-tooth) bioapatite, measurements of carbon isotope compositions yield valuable insights into seasonal changes in paleo-diet and migration patterns ([3] [67] [68]). In case of domesticated animals, where no migration takes place, δ^13^C_ap_ variations indicate changes in (paleo-)diet ([62] [69]).

In seasonally migrating taxa, seasonal stable carbon and oxygen isotope profiles from tooth enamel are expected to be in phase as migration changes the provenance and type of diet as well as the local environment of the animal, affecting both stable isotope proxies synchronously ([37] [70]). However, as shown by earlier studies, in domestic taxa these proxies need not be in phase since a seasonal change in diet can lag or lead the environmental seasonality ([2] [20] [71])

### 2.4 Trace elements in bioapatite

While stable isotope values in bioapatite carbonate have been used for paleo-environmental and paleo-dietary reconstruction, the meaning of trace element concentrations in bioapatite records remains poorly understood ([72] [73]). There is a significant body of work focusing on the post burial incorporation of these elements (e.g. [74] [75] [76]). The incorporation of trace elements in bone and teeth through diagenesis is so prominent that trace element signatures in fossil bone have been proposed as a proxy for fossil provenance ([9] [77] [78] [79] [80] [81]). Though some elements seem to be taken up post mortem by diagenetic processes, calculations of diffusivities show that this pathway does not fully explain trace element abundances found in fossil bioapatite and that a significant portion of the trace element concentration taken up in vivo is retained in fossil bioapatite ([81]). The mechanisms by which trace elements are included in bone and teeth are not well understood and are likely to vary per element ([82] [83]). It has been proposed that trace element concentrations in mammal bioapatite reflect the diet and trophic level of the individual ([9] [74] [84] [85] [86] [87]). Uptake of trace elements through drinking water is regarded to be too low to explain the concentrations found normally in teeth, and it is instead suggested that, in addition to diet, ingested soil and dust accounts for the trace elements incorporated into mammal bone and teeth ([83]). If trace elements found in bioapatite are ingested through food or dust, changes in trace element concentrations are expected to occur through seasonal migration patterns, changes in diet or variation in the availability of dust.

### 2.5 Seasonality in environmental trace elements

Both the amount of airborne dust (airborne particles with a diameter >4 nm and <100 μm, [88]) and its trace element composition vary seasonally (e.g. [89] [90] [91]). These changes could affect trace element abundances in the body of mammals either through direct soil and dust ingestion ([83]) or indirectly through ingestion of drinking water or plants that take up these trace elements from precipitated dust. A peak in the amount of dust deposition in Northern Europe in summer is strongly correlated with peaks in the deposition of trace elements such as Sr ([91]), suggesting that airborne dust is an important source of these trace elements in this area. Combined with large rainfall events in Belgium being concentrated in summer ([92] [93]), a significant increase in the deposition of trace element enriched dust particles occurs in the summer months.

Resulting changes in trace element concentrations in local ground and surface water could influence both the trace element concentrations in drinking water and that of ingested food, especially if the food is grown locally from the same water source used for drinking water. Changes in trace element concentrations in coastal ground water show a seasonal pattern, but besides dust precipitation they are also related to changes in water influx, groundwater level, redox state, pollution and exchange with ocean water ([94] [95]; [96]). Trace element concentrations in surface and ground water, like those of dust particles, are shown to peak in summer ([94]).

The seasonal pattern of these sources of trace elements points towards higher trace element concentrations in the environment of the horse during the summer period ([94]). This seasonality signal can be incorporated into the body fluid of mammals by ingestion either through food or water and incorporated in the teeth. In this case, trace elements are incorporated in larger concentration into dentine and deeper enamel layers through the pulp cavity, causing trace element concentrations to increase with enamel depth ([97]). Trace elements can also be incorporated into the enamel from the outside of the tooth. This post-eruption incorporation would cause a profile of decreasing trace element concentrations with depth ([98] [99] [100]). Both mechanisms are in agreement with depth profiles of several trace elements (e.g. Zn, U and Sr) found by [81] and [83] in modern teeth of domesticated cattle and wild mammal taxa. It is shown that trace element concentration and isotope ratios are more stable in enamel than in dentine, as part of the dentine (secondary dentine) is continuously deposited after initial deposition [101]. Records of seasonal changes in trace element availability preserved in tooth enamel, either during mineralization or post-eruption, therefore constitute promising proxies for seasonal variations in diet or in environmental parameters such as dust availability during tooth formation.

## 3. Materials and Methods

### 3.1 Studied specimen

The teeth used in this study are from a single adult male Belgian draft horse (race: *Brabançon*) kept in the region of Eastern Flanders, Belgium, born in May 2008 and deceased in August 2014. The horse lived outdoors on a cool-season grass pasture (C_3_) situated on the Eocene sand deposits of northern Belgium, allowing it to graze year-round. The fraction of fruit-bearing trees or C_4_ vegetation in the part of the diet ingested by grazing was negligible. Draft horses have an efficient metabolism compared to other races and are usually sustained on a diet consisting almost exclusively on foraged raw food ([102]). Due to their vulnerability to obesity, the proportion of diet consisting of molasses-containing food supplements and starch-rich maize is usually avoided or strongly limited in draft horses (<10% of diet, or ~0.2% of the animal’s weight per day in winter, Prof. Paul Simoens, personal communication; [102]). It can however not be excluded that, besides abundant amounts of hay, the horse’s diet was supplemented with small amounts of concentrated food pellets (oats and barley (C_3_) with added vitamins and minerals usually mixed with molasses from local sugar beet refineries; [103]) and possibly small amounts of maize (C_4_) in the winter season (December, January and February), when fresh grass is less available (Prof. Geert Janssens and Prof. Richard Ducatelle, personal communication).

Drinking water was supplied from local meteoric water and was subject to quality checks of the Flanders Institute for Animal Health (DGZ) as summarized in S7 File. These quality guidelines restrict the maximum concentration of common trace elements to the mg/L level (up to tens of ppm by weight). The animal had been euthanized at Ghent veterinary science department for reasons unrelated to this study. The owner of the animal has given his personal consent for the use of its remains for research and permission for the use of the material was given by Prof. Paul Simoens of UGent’s veterinary science department.

The full upper right row of cheek teeth was removed from the animal and cleaned using cold water maceration ([104]) for 72 hours at 35°C in a SW22 shake bath (Julabo GmbH). The outer surface of cheek teeth was cleaned with milliQ water, abraded superficially with a diamond-coated polishing disk, and left to dry in an oven at 50°C. Cleaning by abrasion was done to rid the surface of the teeth of any varnish or other superficial contamination that was visible and not to produce a smooth surface. The thickness of enamel removed by this process was not visible with the naked eye (< 0.1 mm).

### 3.2 μXRF measurements

μXRF line scans were executed on the cleaned and abraded mesial enamel surface of all cheek teeth (P^2-4^ and M^1-3^). Measurements from the uppermost part of the crown and lowermost part of the enamel, which showed discoloration that was not removed by aforementioned superficial cleaning, were excluded. All line scans were done on a Bruker M4 Tornado μXRF scanner (Bruker, Germany) using a Rh source tube at 50 kV and 600 μA. All XRF analyses were carried out at the XRF lab of the Vrije Universiteit Brussels (VUB, Brussels, Belgium). The X-Ray beam was focused with a polycapillary lens and fluorescence X-Ray spectra were recorded using a Si drift detector. A continuous line of individual points of 25 μm diameter with an integration time of 10 seconds per point were combined into line scans of up to 90 mm. The line-scan method thus produced a straight line of circular points with a diameter of 25 μm. Total measurement time was approximately 10 hours per tooth. During the measurements, teeth were kept horizontal in a container filled with 3 mm glass beads (Carl Roth GmbH). For some teeth, line-scans had to be composed of several line segments to accommodate irregular or sloping tooth surfaces. This way, samples need not be completely flat to allow for reliable measurements, as the X-ray beam could be refocused between line segments. The attenuation length of X-Ray photons (penetration depth after which X-ray intensity drops to 1/e times the original intensity) into bioapatite can be calculated by the Beer-Lambert Law ([105]). It varies with the X-ray photon energy and is therefore different for each element. The attenuation lengths of elements measured in this study range from 10 μm (Mg) to 600 μm (Sr). An overview of the attenuation lengths of X-rays of the different energies associated with analyzed elements into bioapatite is given in S9 File. Point spectra were deconvoluted and quantified using Bruker Esprit software and the errors of deconvolution introduced in the conversion of spectra of XRF counts to trace element concentrations (hereafter: deconvolution errors) were calculated using Bruker’s ARTAX spectral analysis software ([31]). All measured concentrations that were below a detection limit of three standard deviations of deconvolution were rejected. Correction factors needed to compensate for matrix effects in the X-Ray Fluorescence functional parameters quantification algorithm were determined for all elements using one-point calibration with the ISO certified BAS-CCB01 bioapatite standard (Bureau of Analysed Samples Ltd., Middlesbrough, UK, for certified values please see S8 File). All elemental concentrations were recalculated from mass to molar percentage and trace element concentrations were divided by the concentration of calcium and given in mmol/mol. Individual μXRF point measurements were checked based on P/Ca ratio. All points with a P/Ca ratio deviating more than one standard deviation from the mean P/Ca value were rejected. The Si concentration was higher on the edges of the teeth where Si was measured in the glass beads supporting the teeth during the measurement, driving the average of all Si measurement to an (for bioapatite samples) unreasonable value of 4%. Close observation of the data showed that the transition of measurements from the glass beads to the sample was sharp and took place in about 15 measurements (375 μm). A conservative threshold value of 5% was chosen to reject measurements that were contaminated by the glass beads while avoiding the removal of bioapatite measurements. Measurements with Si concentrations higher than 5% were rejected. Repeated point measurements (N = 30) on the BAS-CCB01 cremated bone bioapatite standard were used to calculate repeatability standard deviations (hereafter: measurement errors) of μXRF measurements. The BAS-CCB01 standard is chosen for having same matrix as the samples that were measured (bioapatite), allowing the error of matrix effects in the XRF measurements to be included in the reproducibility testing.

### 3.3 Isotope ratio mass spectrometry

Samples for stable isotope analysis were drilled on the same mesial side of the teeth as used for μXRF analyses, using a dental drill with a diamond coated drill bit. About 40 mg of enamel powder was collected from lines with an average width of 0.8 mm and length of 20 mm drilled perpendicular to the growth axis of the tooth. Care was taken to exclusively sample the enamel layer as the dentine has a larger organic matter content and may therefore have a different isotope signature ([106] [107]).

Samples for stable isotope analysis were subject to a conservative pretreatment in order to facilitate comparison of stable isotope results with those of archaeological and palaeontological studies, where pretreatment is necessary. Approximately 11 mg of sample were pre-weighed for pretreatment and subsequently treated with a 1 M calcium acetate-buffered acetic acid (CH_3_COOH) solution in excess for 30 minutes ([108]). After treatment, samples were rinsed three times with milliQ water and dried overnight at 50°C. Dried pretreated samples were weighed to determine weight loss during the pretreatment procedure. Compared to conventional pretreatment procedures (e.g. [109]), acid treatment time was shortened and no agent for organic matter removal was used. This decision was made based on results from multiple studies showing that care should be taken in applying conventional pretreatment methods (especially using long reaction times) and that the use of oxidizing agents for the removal of organic matter in enamel is likely to be superfluous and might introduce error in stable isotope measurements ([108] [110] [111] [112]).

On average 1.4 mg of pretreated sample was weighed for stable carbon and oxygen isotope (δ^13^C_ap_ and δ^18^O_c_) measurements of the carbonate fraction on a Nu Perspective Isotope Ratio Mass Spectrometer (IRMS) with a NuCarb carbonate preparation device in the stable isotope lab of the Vrije Universiteit Brussel (VUB, Brussels, Belgium). Samples of ±1 mg of bioapatite were reacted for 10 minutes with phosphoric acid (H_3_PO_4_.H_2_O) at 70°C and produced CO_2_ was led into the mass spectrometer using a dual inlet device. Mass spectrometry results were corrected for variations in the amount of produced CO_2_ and instrumental drift and then corrected using a three point calibration with the in-house MAR2 carbonate standard (δ^13^C: 3.41‰ ± 0.10‰; δ^18^O: 0.13‰ ± 0.20‰, calibrated using the NBS-19 standard; [113]), the in-house Enf enamel standard (δ^13^C: -9.83‰ ± 0.08‰; δ^18^O: -5.41‰ ± 0.30‰) and the in-house CBA calcine bone standard (δ^13^C: -14.77‰ ± 0.18‰; δ^18^O: -9.97‰ ± 0.21‰). The Enf and CBA standards were calibrated using NBS18, NBS19 and IA-R022 Calcium Carbonate (Iso-Analytical Ltd, Crewe, UK; δ^13^C: -28.63‰ ± 0.09‰; δ^18^O: -22.69‰ ± 0.11‰). Repeatability of independent MAR2 measurements (N = 68) yielded a standard deviation of reproducibility of 0.07‰ and 0.08‰ for δ^13^C_ap_ and δ^18^O_c_, respectively. All isotopic values are reported relative to Vienna Pee Dee Belemnite (VPDB). Carbonate content was calculated from sample weight and CO_2_ pressure, using a linear relationship observed between CO_2_ pressure in the dual inlet and weight of pure carbonate (MAR2) samples. All samples with insufficient CO_2_ production for a reliable stable isotope measurement were rejected. The standard deviation of reproducibility of CO_3_ content measurements was 0.50% for the ENF standard (N = 29) and 0.12% for the CBA standard (N = 30). Stable isotope records were compared to monthly mean temperature data measured in Vlissingen (Netherlands), obtained from the open database of the Royal Netherlands Meteorological Institute (KNMI, Netherlands).

## 4. Results

### 4.1 μXRF line scanning

Table 1 shows that the deconvolution errors (referred to as machine errors) are lower than the measurement errors calculated from repeated measurements on the BAS-CCB01 standard (measurement errors). These repeated measurements show that only Na, Mg and Ni have standard deviations above half their mean value. Therefore, the values obtained for these elements are not statistically significant (statistically separable from zero) within a 95% confidence level of two standard deviations of measurement error. Correction factors determined using the BAS-CCB01 standard show large variations between elements. Relatively low measurement errors show that the method yields reproducible results for elements heavier than Mg with concentrations above 1 ppm. Accuracy for these elements was <5% (for CRM01 certified values see S8 File).

**Table 1:** Table showing the standard deviations of deconvolution (machine errors) and reproducibility (measurement errors) of elemental abundances measured with the μXRF, as well as correction factors implemented to correct the values using the BAS-CCB01 standard (see S8 File).

Element	Mean value:	Machine error	Measurement error	Correction factor
**O**	**62.41%**		**2.06%**	**1.00**
**Ca**	**20.74%**	**38.4 ppm**	**0.67%**	**0.99**
**P**	**16.40%**	**25.3 ppm**	**0.49%**	**2.88**
**Si**	**0.24%**	**6.81 ppm**	**0.01%**	**1.86**
**Na**	**0.03%**	**4.05 ppm**	**0.09%**	**2.11**
**Mg**	**0.13%**	**10.5 ppm**	**0.10%**	**4.50**
**Al**	**108 ppm**	**0.74 ppm**	**22.1 ppm**	**0.19**
**Fe**	**64 ppm**	**0.64 ppm**	**3.80 ppm**	**0.62**
**Zn**	**68 ppm**	**0.29 ppm**	**3.07 ppm**	**1.00**
**Sr**	**183 ppm**	**1.21 ppm**	**2.24 ppm**	**0.84**
**Cu**	**6.0 ppm**	**0.50 ppm**	**1.61 ppm**	**1.00**
**Ni**	**0.3 ppm**	**0.01 ppm**	**0.5 ppm**	**1.00**
**Ba**	**61 ppm**	**1.10 ppm**	**7.11 ppm**	**5.00**
**Pb**	**7.7 ppm**	**0.84 ppm**	**0.74 ppm**	**1.00**
**Ti**	**3.6 ppm**	**0.02 ppm**	**0.98 ppm**	**0.25**
**Cr**	**7.7 ppm**	**1.02 ppm**	**2.05 ppm**	**1.00**

**Fig 1:**
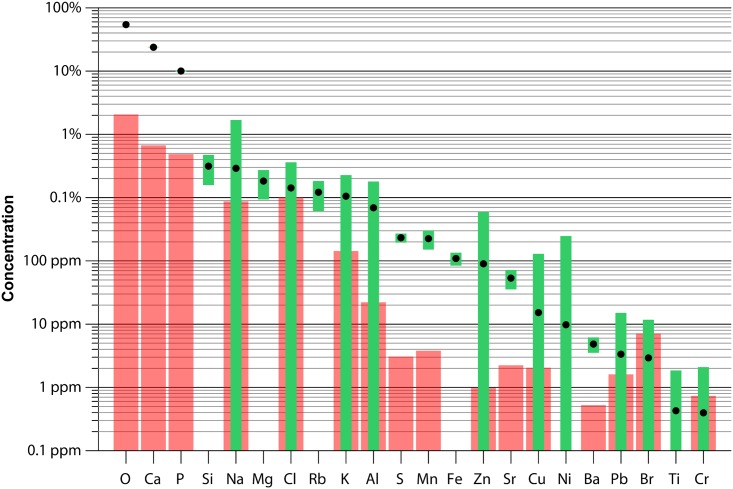
Overview of mean molar concentrations (black dots) and concentration ranges (green areas) of all elemental abundances detected and quantified with μXRF. Red bars show standard deviations of reported reproducibility tests on the BAS CCB01 standard (see Table 1). Not all elements have red bars because concentrations of only a few elements were certified for the BAS-CCB01 standard. Note that red bars in this Fig are standard deviations on the BAS-CCB01 standard, while means and ranges of concentrations indicated in green are from the horse bioapatite.

Fig 1 shows average values and ranges of elemental abundances measured on the bioapatite after excluding points with deviating Ca/P ratio and high Si concentrations (see above). Major elements are present in a Ca:P:O molar ratio of 10: 4: 20. The most abundant trace elements are cations (e.g. Mg, Rb, Sr, K, Al), while Cl and S are also present in relatively high abundance. Concentrations of trace elements Zn, Sr and Ba are ~300 ppm, ~100 ppm and ~30 ppm respectively. Relatively high concentrations are found for the so-called “bone-seeking elements” Zn, Sr, Ba and Pb as well as other common trace elements, such as Na, Mg, Cl and K. Concentrations of Si, Mn and Al are 0.3%, 200 ppm and 0.07% respectively.

Lighter elements show more variation and have higher measurement errors than elements heavier than Al (Fig 1). Values for Ti and Ni show a very large range, and a large portion of the values for these elements are below 1 ppm. A large part of the Ni, Ti and Cr concentrations is below the measurement error, and is not statistically significant. Therefore, even though mean values of these elements are above the confidence level of two standard deviations of measurement error, most results of Ni, Ti and Cr were not reliable enough for the records to be interpreted. Measurement errors for other more abundant trace elements (Table 1) are between 1% and 5% of the measured value.

### 4.2 Stable isotopes and pretreatment

**Fig 2:**
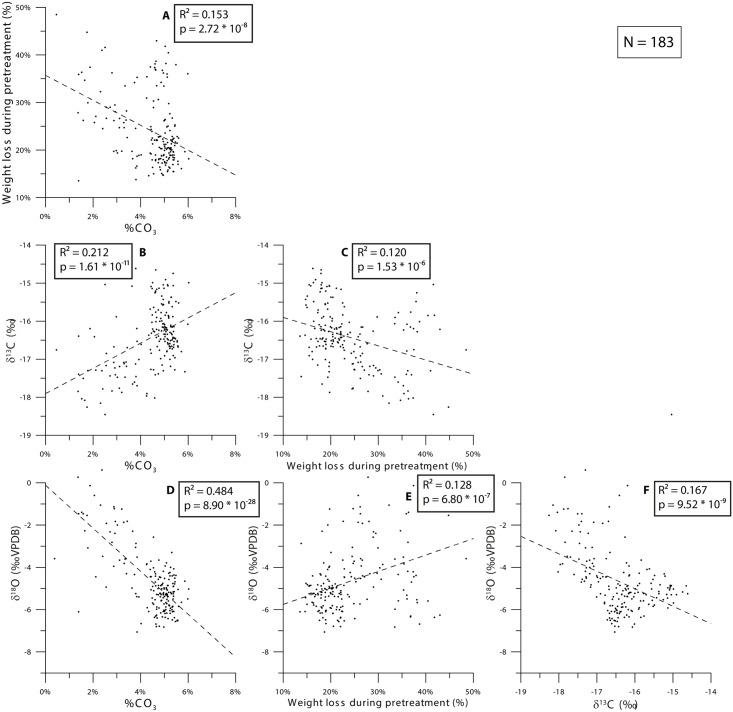
Cross plots of all combinations of the four parameters obtained by pretreatment and mass spectrometry: carbonate content (%CO_3_), weight loss during pretreatment (%), carbon isotope composition (δ^13^C_ap_) and stable oxygen isotope composition (δ^18^O_c_). Dashed lines show linear regressions through the cross plots and p-values and coefficients of determination (R^2^) of the regressions are given.

Mass spectrometry results (Fig 2) show that δ^13^C_ap_ varies between -19‰ and -13‰. δ^18^O_c_ ranges between -7‰ and 0‰. Weight loss caused by the pretreatment procedure ranges between 14% and 48% (Fig 2A, 2C and 2E). Carbonate content of enamel samples exhibit a variation between 1% and 6% (Fig 2A, 2B and 2D).

Linear regressions between all measured parameters have low (<0.01) p-values indicative of a significant linear trend in the data. The R^2^ values calculated for these linear trends are below 0.25 for all regressions except for the regression between δ^18^O_c_ and carbonate content. With a coefficient of determination (R^2^) of 0.48, stable oxygen isotopes show a weak negative relationship with carbonate content (Fig 2D). Fig 2F shows that there is no correlation between the two stable isotope proxies, and Fig 2C and 2E show that there is no linear correlation between the loss of sample weight in the pretreatment procedure and measured isotope ratios. Weight loss by pretreatment shows no significant correlation with carbonate content in the sample after pretreatment (Fig 2A).

### 4.3 Tooth records

**Fig 3:**
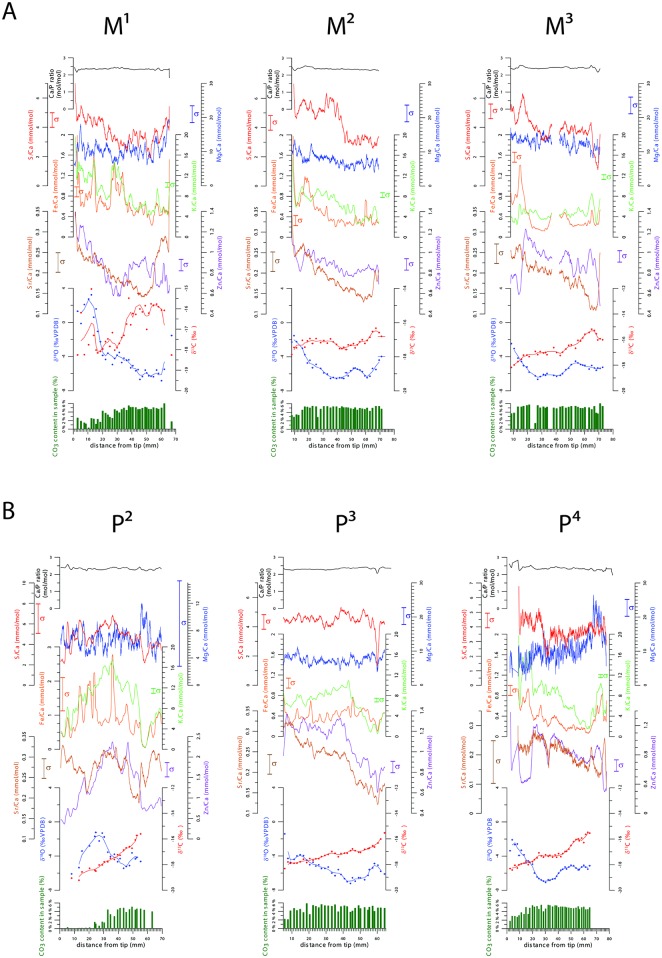
Records of carbonate content (green bars), stable isotope compositions (δ^13^C_ap_ in red and δ^18^O_c_ in blue) and trace element ratios (dots, colored lines represent moving averages) of all molars (A) and premolars (B). Larger vertical bars indicate the specific measurement error for each tooth and each element. The black line on top shows how Ca/P ratios vary over the line-scan. Vertical error bars plotted on the inside of y-axes mark the tooth- and element-specific measurement errors (σ).

A selection of trace elements with relatively high abundances in the studied teeth (Sr, Zn, Fe, K, S and Mg) is discussed in terms of variations through time in the measured sample. Results for individual teeth of the modern horse show the full potential of μXRF for high resolution trace element abundance line scanning (Fig 3A and 3B). These figures also document the spread of data between individual points as a result of small variations in surface conditions and concentrations in the sample. Larger irregularities on the sample surface were accommodated by refocusing the X-ray beam between line segments. In order to visualize the mm-scale trends of XRF records through the tooth length, a 50 point moving average was constructed by averaging, for every point that was measured, the measured value on this location with values of 49 measurements directly above and below the measurement. The smoothed records thus obtained illustrate that mm-scale variations in the records of trace element concentration are larger than the measurement error, and therefore statistically significant.

The measurement error (shown on Fig 3A and 3B as vertical error bars) varies between different elemental ratios and between different teeth depending on the surface conditions, and is relatively high for elements with a lower abundance, such as Sr (see also Fig 1 and Table 1). Fig 3A and 3B show that the record of Mg, which has a relatively high abundance, contains considerable spread with various high amplitude mm-scale shifts. These mm scale shifts in the Mg/Ca 50 point moving average seem to correlate to mm-scale shifts in other records of element ratios, but have a larger amplitude in Mg/Ca. Mg/Ca records are shown for all teeth except for P^4^, where the Mg peak was too small to produce a continuous record. These Mg/Ca records show that deconvolution of an insignificant XRF peak produces a record with large (artificial) mm-scale shifts in abundance.

For paleo-environmental reconstruction purposes, the larger-scale variations that occur on a spatial resolution in the order of multiple centimeters are more interesting. On this scale large shifts in elemental abundance take place, which are significant with respect to the reported measurement errors. Comparison with Mg/Ca records also shows that the larger cm-scale variations observed in other trace element records are not present in Mg/Ca. Fig 3A and 3B show that most cm-scale variations in trace element ratios are of the same order of magnitude through different teeth (M^1-3^ and P^2-4^). Exceptions are the K/Ca and Zn/Ca ratios of P^2^ and the Fe/Ca ratio of P^3^, which are plotted on a separate scale to illustrate the variations observed in these records. The K/Ca and Zn/Ca values as well as other trace elemental concentrations in P^2^ are higher than in other teeth. The Fe/Ca values from P^3^ are lower than in the other tooth records.

Fig 3A and 3B show that carbonate content in the individual tooth records is always lowest in the oldest part of the tooth, closest to the tip. These low values for carbonate content coincide with high δ^18^O_c_ values in all teeth except for P^2^. The tip of most teeth is also associated with low δ^13^C_ap_ values, although the trend is less pronounced than in the δ^18^O_c_ record. Samples with lower carbonate content further down the teeth do not show higher δ^18^O_c_ values or lower δ^13^C_ap_ values. The oldest 15–20 mm of the M^1^ δ^18^O_c_ record shows the highest values of all records, exceeding 0‰ (Fig 3A). The cm-scale trend observed in the δ^18^O_c_ of M^1^ is increased by these high δ^18^O_c_ values but is still visible if these samples are removed from the record.

The spatial resolution of stable isotope measurements is not good enough to show mm-scale variations in the teeth, but cm-scale trends are observed. Comparison on centimeter scale shows that trace element records and oxygen isotope records show a similar trend, with trace element records shifted horizontally by approximately 20 mm compared to oxygen isotope records. The Sr concentration record seems to be more in phase with the δ^18^O_c_ record than the other trace elements, but still lags the isotope record by about 10 mm. To compare and interpret variability in the multiproxy records from horse teeth, multiple teeth from the individual are combined to create an age model.

### 4.4 Age model

**Fig 4:**
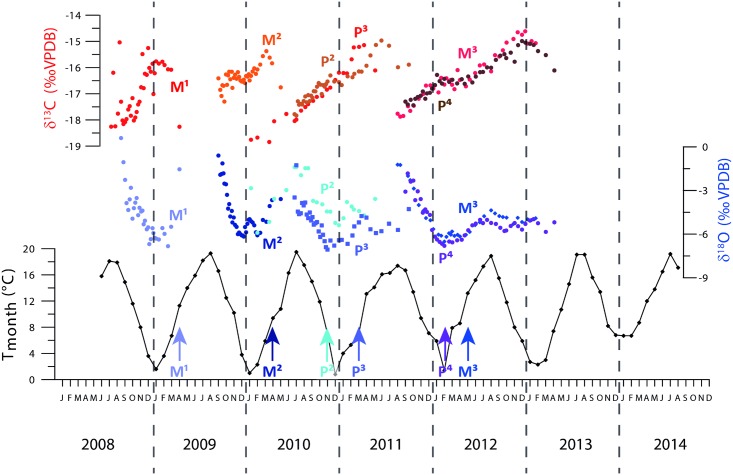
Stable oxygen (blue to purple) and stable carbon (red to brown) isotope records of all teeth of the modern horse correlated to a local monthly mean temperature curve (Vlissingen, Netherlands, Royal Dutch Meteorological Institute). Dots represent individual measurements. The standard deviation of reproducibility is contained within the dot. Different colors are associated with different teeth. Tooth records are labeled in the same color as their measurements. Arrows (in the same color as stable oxygen isotope measurements) indicate the approximate eruption times of the teeth according to [34]. The age model used for the remainder of the study is based on these eruption patterns and on the linkage of δ^18^O_c_ record to the temperature record using a positive correlation between the two.

Using the eruption pattern of horse teeth ([34] [42]), stable isotope plots for individual teeth can be superimposed on the monthly mean temperature record (Fig 4). A first tentative age model based on eruption times of the teeth shows that the oxygen isotope record lines up with the monthly temperature record. This shows that a positive relationship between precipitation, seasonal temperature and stable oxygen isotope ratios in mammal teeth, as proposed in other studies ([3] [114]), is valid in this study as well. Maxima and minima in both δ^18^O_c_ and temperature are used as additional tie points to constrain the age model. According to the age model, tooth mineralization occurred over a period of 4–5 years, which is in agreement with [34] This age model is applied on the isotope data as well as on the trace element records.

### 4.5 Seasonal variations

**Fig 5:**
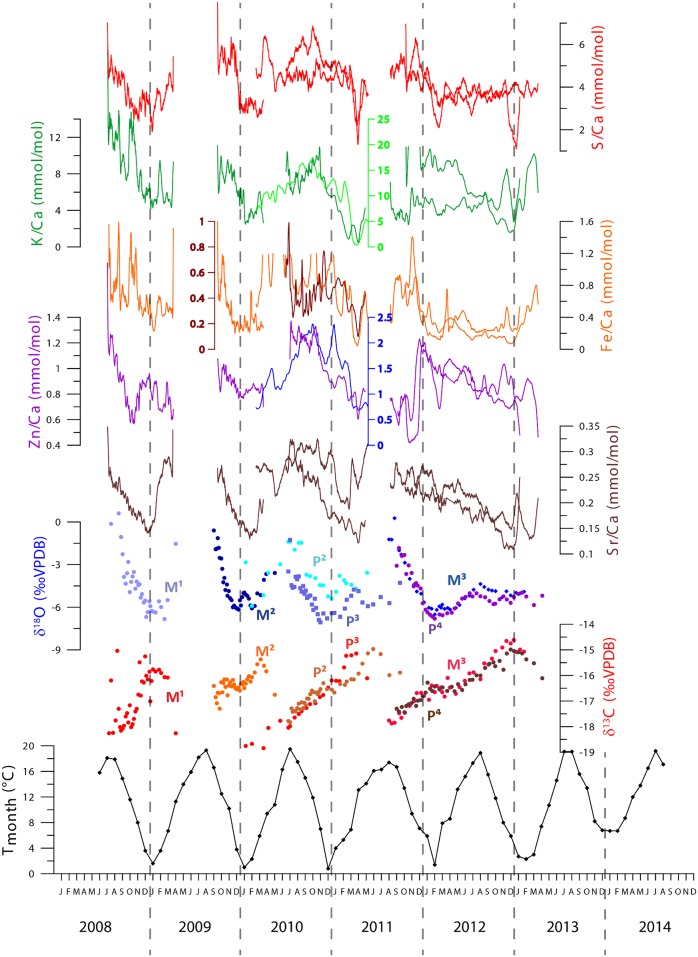
Records of stable isotope ratios and trace element ratios plotted against time using the age model based on the relationship between oxygen isotopes and temperature seasonality. Dots represent individual stable isotope measurements. The standard deviation of reproducibility of these measurements is contained within the dot. Different colors are associated with different teeth. Tooth records are labeled in the same color as their measurements Note that three of the trace element records are plotted on a different scale (see also Table 2). These three trace element records are shown in a different color and their scales are shown to the side of the record in that same color.

Stable carbon isotope ratios measured in horse enamel seem to be in antiphase with respect to oxygen isotopes, even though there is no linear correlation between the two proxies (Fig 2). A seasonal cyclicity is observed in δ^13^C_ap_ values with lower δ^13^C_ap_ values in the summer and higher δ^13^C_ap_ values in the winter season. When all trace element records are plotted on the same time axis using the age model, a clear seasonal pattern emerges in all plotted records (Fig 5). It must be noted that the K/Ca and Zn/Ca records of P^2^ and the Fe/Ca record of P^3^ are plotted on individual scales, but they follow the same seasonal pattern. Patterns in both oxygen and carbon isotope ratios measured in different teeth mineralizing in the same time period (e.g. P^2^/P^3^ and P^4^/M^3^) show a very similar pattern. Overlapping μXRF records (plotted as different lines in Fig 5) match as well, even on small spatial scales. The cm-scale annual cyclicity in the trace element ratios is best expressed in the middle part of the record, where records from multiple teeth overlap and more tie points are available to create a more reliable age model. Towards the oldest and youngest parts of the composite record the age model has to be extrapolated resulting in a slight mismatch of elemental abundance records with the monthly temperature record. To illustrate differences in the seasonal extent of trace element composition between individual teeth, seasonal ranges and annual averages of all tooth records are shown in Table 2. The offset of trace element values in P^2^ and P^3^ with respect to other records is not similar (e.g. variation and absolute values are higher than average in P^2^, but lower in P^3^). Seasonal variations in S/Ca, Sr/Ca and both stable isotope proxies are of the same order of magnitude in all teeth.

**Table 2:** Summary of the seasonal variation observed in all trace element records from all teeth and the observed annual average. Trace element concentrations indicated in orange (K/Ca and Zn/Ca of P^2^ and Fe/Ca of P^3^) have different values from the same records in other teeth.

Elemental ratio	Values in mmol/mol	P^2^	P^3^	P^4^	M^1^	M^2^	M^3^
S/Ca	min value	2	4	2	2	2.8	1.8
	max value	7	5	4.5	5.5	6	6
	mean value of cycle	**5**	**4.5**	**3.5**	**3.5**	**4.5**	**4**
K/Ca	min value	2	1	1	4	3	2
	max value	16	10	12	13.5	10	9
	mean value of cycle	**10**	**6**	**7**	**9**	**6**	**5**
Fe/Ca	min value	0.2	0.1	0.2	0.2	0.25	0.1
	max value	1.5	0.6	1	1.3	1.2	1.3
	mean value of cycle	**0.8**	**0.4**	**0.5**	**0.8**	**0.7**	**0.6**
Zn/Ca	min value	0.5	0.7	0.4	0.5	0.75	0.6
	max value	2.4	1.3	1.2	1.2	1.1	1.2
	mean value of cycle	**1.5**	**0.9**	**0.7**	**0.9**	**0.9**	**0.8**
Sr/Ca	min value	0.2	0.15	0.12	0.14	0.13	0.12
	max value	0.32	0.32	0.28	0.28	0.27	0.27
	mean value of cycle	**0.26**	**0.22**	**0.2**	**0.22**	**0.2**	**0.19**

## 5. Discussion

### 5.1 Pretreatment and carbonate content

The lack of correlation of weight loss during pretreatment with CO_3_ content as well as with stable isotope results shows that the amount of material removed from the samples during pretreatment does not influence the resulting stable isotope ratios and measured carbonate contents in a systematic way. The observation of seasonal patterns in stable isotopes and the fact that all isotopic values fall within the expected range for modern teeth indicates that contamination of the samples after pretreatment is unlikely, or at least that it has no effect on the interpretation of these seasonal cycles.

Fig 3A and 3B shows that carbonate content is lowest in the oldest, most mature parts of the enamel of all teeth. This can be explained by a decrease in carbonate content in older enamel related to its maturation process ([115]). Higher δ^18^O_c_ and lower δ^13^C_ap_ values are observed in the oldest parts of all teeth except P^2^. This exception and the fact that carbonate content in younger parts of tooth records does not correlate with δ^18^O_c_ and lower δ^13^C_ap_ shows that the relationship between stable isotopes and carbonate content is only valid for samples from the tips of the teeth. Since all reported stable isotope measurements were corrected for variations in produced CO_2_ during the acid reaction and all samples yielded enough CO_2_ for measurement, fractionation during the measurement cannot account for the observed correlation. Such a fractionation would also have driven δ^18^O_c_ and δ^13^C_ap_ values in the same direction ([116] [117]), while observations show that the correlation with carbonate content is negative for δ^18^O_c_ values and positive for δ^13^C_ap_ values. The correlation with stable isotope values is instead driven by the fact that all teeth except P^2^ started mineralizing in the summer season and therefore have high δ^18^O_c_ at their tip. As a consequence, we argue that variations in carbonate content are a result of tooth development, while stable isotope ratios vary according to environmental and dietary parameters.

### 5.2 Seasonality in stable isotope ratios

A seasonal range of 3‰ observed in carbon isotope ratios is much smaller than that found in studies of mammal enamel in which a seasonal change in diet from C_3_ to C_4_ vegetation is suggested ([3] [20] [118]), in which case a seasonal range of 6‰ was reached. The range obtained is in good agreement with seasonal shifts of 3‰ found in horse enamel by [13], which indicate comparatively small annual variations within a diet composed almost exclusively of C_3_ vegetation. The mean of δ^13^C_ap_ value of -16‰ is also in agreement with a diet consisting exclusively of C_3_ vegetation ([35] [119]). This is to be expected from a domestic horse fed on a constant diet of fresh grass supplemented with small amounts of cereal grains. The 3‰ seasonality in δ^13^C_ap_ indicates that there were small seasonal changes in diet through the year. Such a change resulted from an increase in grazing in summer while in winter a larger part of the diet consisted of dry food. A larger relative proportion of grass at the expense of dried cereal (e.g. barley) would explain more negative δ^13^C_ap_ values in enamel in summer, since grass has a more depleted δ^13^C signature ([63]). Incorporation of minor amounts of maize (a C_4_ plant) into the dry food that made up to winter diet probably explains the higher (less negative) carbon isotope signature in this season. Several factors, such as water availability, amount of sunlight and growth rate, can also cause seasonal changes in the δ^13^C value of plants ([120] [121]). These factors could change the δ^13^C value of grass consumed by the individual and amount to part of the seasonality observed in δ^13^C_ap_ values of horse teeth.

Oxygen isotope values reported here exhibit a seasonal range of 4–5‰, which is similar to the seasonal variation observed in horse teeth from mid-latitude setting of North Dakota by [13]. Ranges are larger than the 2‰-3‰ found in studies of sheep from Orkney Island ([3] [118]) and pig teeth from Corsica ([20]). Variation in seasonal δ^18^O amplitude of the rainwater on these different locations explains the observed difference in seasonal amplitude of δ^18^O_c_ between this study and those mentioned above. The sheep and pig teeth originate from island specimens where marine influence may lower the seasonal amplitude of the isotope composition of precipitation compared to more terrestrial environments ([58]). The horse in the present study lived at a distance of ~60 km from the North Sea coast and ~450 km from the Atlantic Ocean, it is expected that marine influence on the continental mainland will presumably be less important than on an island setting. Indeed, the seasonal amplitude of rainwater in coastal Northern Europe is approximately 4‰ ([57]; Keven De Bondt (VUB), personal communication) Another explanation for the difference in seasonal amplitude might be that differences in the uptake of water or the formation of the teeth between species cause differences in seasonal amplitude in oxygen isotopes between sheep, pig and horse teeth. Absolute δ^18^O_c_ values are in good agreement with seasonal variations in oxygen isotopes from horse enamel reported by [13] and are also in the same order of magnitude of δ^18^O_c_ values found in sheep enamel by [3] and [118] and in pig enamel [20]. Furthermore, oxygen isotope ratios measured in this study are enriched by approximately 3‰ with respect to local meteoric water, which is in agreement with [52]. This shows that the δ^18^O_c_ values obtained are reasonable for modern and archeological mammal teeth, and that the interpretation of cyclic variations in stable isotope profiles as seasonal cyclicity is valid. The highest δ^18^O_c_ values observed in the oldest part of the M^1^ record are most likely caused by effects of weaning in the first months of the animal lifetime. Weaning can increase the δ^18^O_c_ values of tooth enamel in juvenile mammals by as much as 2–3‰ ([54]), and a similar offset in δ^18^O_c_ values is observed in this study. While feral horses wean their young after 8–9 months depending on the race, weaning of domestic foals typically occurs between 4–6 months after birth ([122]). Fig 5 shows that increased δ^18^O_c_ values in the first 15–20 mm are consistent with a weaning period of 4–6 months. Measurements of δ^18^O_c_ values from the tip of M^1^ that are exceeding 0‰ are for this reason excluded from the age model graphs and from further seasonality interpretation (Fig 4).

The approximate antiphase relationship between δ^13^C_ap_ and δ^18^O_c_ values is opposite from the in-phase pattern found by [13], but is in agreement with other studies ([20] [118]). The latter explain seasonal variation in δ^13^C_ap_ values of 6‰ as a result of changes in diet with incorporation of C_4_ plants or fruits in winter, which occurred in this individual only to a small extent. The low δ^13^C_ap_ values observed here are indicative of a diet consisting primarily of C_3_ plants, leading to the assumption that changes in δ^13^C_ap_ observed in these horse teeth are caused at least partly by changes within a C_3_ diet. The 3‰ seasonality in δ^13^C_ap_ observed by [13] in North Dakota is attributed to seasonal aridity affecting the water use efficiency (WUE) of plants in the animal’s diet ([123]). In regions with seasonal aridity, reduced WUE diminishes the carbon isotope discrimination and results in heavier δ^13^C values in drier summer months ([123] [124]). However, the horse studied here lived in a coastal temperate climate with limited seasonal drought and the WUE of the grass is most likely not a leading factor driving its δ^13^C values. It is more likely that δ^13^C_ap_ seasonality is primarily driven by small changes in the composition of the diet, and that changes in δ^13^C of the ingested plants (grass) explain only a small fraction of the carbon isotope seasonality. Since the exact composition of the supplemented winter food and the relative proportion of isotopically heavy maize in the diet is unknown, no reliable mass balance for carbon isotopes could be made for this study. An actual culture experiment in which food and water sources are carefully controlled may shed more light on the effect of dietary supplements on carbon isotope. Such a study would, however, be expensive and time consuming and is beyond the scope of this paper. It cannot be excluded that added maize in winter drove the shift to heavier carbon isotope values in the enamel studied here. The fact that the maxima in δ^13^C_ap_ are recorded during the late winter season (between January and March) shows that the dietary change towards relatively higher amounts of dry food (including C_4_ maize with higher δ^13^C values) is recorded within 2 months (December–February). Such a lag can be explained by the combined effects of the response time of body fluid in mammals (15 days to a month; [13]) and time lag introduced due to mineralization of the teeth (1–2 months; [21] [34] [69]). The seasonal shift in oxygen isotopes is expected to start earlier, as stable oxygen isotope values of rainwater start to drop significantly as early as October ([57]). A phase lag of 2 months of stable carbon isotope seasonality relative to the seasonality of stable oxygen isotopes can therefore be explained by a delayed change in diet.

### 5.3 Reliability of μXRF results

The large variation in correction factors required to calibrate the quantification of μXRF measurements for bioapatite shows that the matrix effect in apatite samples has a big impact (Table 1). This demonstrates that it is always necessary to do a standard calibration specifically for the matrix of the samples that are analyzed, and that failure to do so results in large inaccuracies in the quantified values. The deconvolution error found for μXRF data using the ARTAX software is a gross underestimation of the real measurement error determined by reproducibility tests (Table 1). This shows that it is always necessary to calculate the measurement error on a certified standard with the same matrix composition as the samples (in this case: the BAS-CCB01 bioapatite standard) to determine the standard deviation on the result. Measurement errors obtained for elements heavier than Mg and with a concentration >1 ppm (1 to 5% of the measured value) are comparable with standard deviations reported for LA-ICP-MS and ICP-OES ([125] [126] [127] [128]).

The comparison of elemental records with the Mg/Ca record shows that deconvolution of small XRF peaks leads to noisy records with artificial mm-scale variations. Similar small-scale fluctuations superimposed on the larger seasonal trend of records of other trace elements (Fig 3A and 3B) may also be attributed to errors in the deconvolution of XRF spectra. The larger cm-scale variations observed in horse enamel trace element records represent real changes in chemical composition of the teeth. They reveal a seasonal pattern in trace element ratios in horse enamel that is in antiphase with δ^13^C_ap_ and shows a 2–3 months phase lag with respect to the δ^18^O_c_ values and monthly temperature records.

### 5.4 Trace element abundances in horse teeth

Observed Ca:P:O proportions obtained by μXRF are in agreement with the formula of bioapatite, (Ca, Na, Mg, Ba, Fe, Sr, Zn, []) _10_(PO_4_, HPO_4_, CO_3_)_6_(OH, F, Cl, CO_3_, O, H_2_O, []) _2_, in which part of the PO_4_ and OH groups are substituted by CO_3_. The spread in both Ca and P concentration is low, showing that measurements on the tooth surface sampled almost exclusively bioapatite. Fig 3A and 3B furthermore show that the Ca/P ratio remains relatively constant through all line scans. The most abundant trace elements found in horse teeth are cations substituting for Ca, although Cl and S are also present in relatively high concentrations (>0.1%, see Fig 1). Zn, Sr and Ba are present in the same order of magnitude as those found in other studies of trace elements in enamel ([83]). These results are in agreement with other studies reporting relatively high concentrations of so-called “bone-seeking elements” Zn, Sr, Ba and Pb ([81]) as well as other elements found commonly in bioapatite, such as Na, Mg, Cl and K ([9]). Elemental concentrations that do show a significant offset from values reported in other studies include Mn and Al, which are higher in this study compared to earlier work ([9] [83]). An enrichment in Mn and Al may be a result of different provenance of the animal or a difference in preferential enrichment of the abovementioned elements in horse enamel compared to the other taxa studied in [9] and [83]. A possible source of Mn or Al might be airborne dust, but concentration data from other specimens and their environment would be needed to pinpoint the source of these elements in horse enamel and determine why concentrations are elevated in the current specimen.

Some small mm-scale variations in trace element records are repeatable through different teeth and are probably linked to small weekly to monthly scale variations in growth rate or enamel thickness ([129]). These differences in enamel growth rate can influence trace element concentrations ([34]). Since the integration depth of the μXRF remains constant for each given element (see Methodology section), variations in the enamel thickness can result in variations in the trace element concentrations measured when these concentrations change with depth in the enamel ([83]).

### 5.5 Seasonality in trace elements

The consistency between different trace element records suggests a single mechanism for in vivo uptake of all reported trace elements into the enamel. The seasonal cyclicity observed in all records suggests that the variation in trace element concentrations in teeth is driven by environmental and/or dietary factors rather than by internal changes in the trace element uptake mechanism during enamel formation. It has been suggested that a large part of the trace elements is acquired in mammal tissues from environmental sources and therefore vary with the rate of environmental uptake ([87] [130]).

The use of a domestic horse in this case effectively rules out changes in trophic level or migration as drivers for trace element variations in teeth. An increase in grass consumption at the expense of cereal grains, hay and maize, indicated by the small but significant seasonal variation in δ^13^C_ap_ values, shows that a small seasonal change in diet could explain the seasonality in trace element ratios as well as carbon isotopes, especially since trace element and δ^13^C_ap_ records are almost exactly in antiphase (Fig 5). A seasonal change in the intake and composition of soil and dust constitutes an additional mechanism that could explain the observed seasonal pattern in trace element ratios (as suggested in [83]).

Both trace element concentrations in dust particles in the atmosphere and abundance of these particles in Europe are highest in the summer period and could lead to the observed seasonal signal in enamel trace elements for several months ([89] [91]). Such an increase in trace element deposition through dust particles provides a possible explanation for the higher concentration in trace elements in horse teeth in the months directly following summer. Dust could enter the animal by direct ingestion ([83]) or indirectly through uptake in local plants and surface water ([131]). The latter pathway is plausible, since trace elements dissolved in surface water peak in summer ([94]) just like trace element concentrations found in enamel in this study. Furthermore, the trace element concentrations of drinking water were disregarded in earlier studies on the basis of them being too low to affect the concentrations of body fluids in mammals ([83]). The quality control mentioned in the Materials and Methods section further restricts the trace element concentrations of drinking water to values much below the values measured in tooth enamel (see S7 File). Seasonal variations within these low concentrations (ppm level) would have little effect on the larger changes in trace element concentrations in tooth enamel. We therefore reject drinking water as the source for trace elements in horse enamel and assume that trace element ingestion through dust and/or changes in diet explain a large part of the seasonality in trace element ratios in enamel found in this study.

The observed phase lag of trace element records of 2–3 months with respect to the seasonal oxygen isotope signal could indicate that the total lag between trace element uptake and incorporation into the tooth enamel was 2–3 months slower than that of oxygen isotopes. Such a lag could be explained by a longer residence time of bone-seeking trace elements in the body of horses compared to that of lighter and more abundant oxygen and carbon atoms ([132]). The response time of body fluids suggested as a source of time lag in the uptake of carbon and oxygen isotopes ([13]) might be longer for trace elements. The lower concentrations of these elements in the animal’s body or their larger atomic weight may explain a longer residence time, causing a time lag in the apparent seasonal signal (as in [13]). Another explanation might be that the moment of uptake of trace elements lags the temperature seasonality and that the highest concentrations of trace elements are actually taken up 2–3 months later than the oxygen isotopes (near the end of summer). A lag of 2–3 months due to reservoir effect in the horse body on trace element incorporation into horse tooth enamel would place the moment of ingestion of highest concentrations of trace elements in the middle of the summer (in phase with high δ^18^O_c_). If the trace element seasonality is caused by changes in diet, this would suggest that diet in summer would be more enriched in trace elements than in winter. The fact that trace element seasonality is in antiphase with δ^13^C_ap_ seems to suggest such a relationship and favors the hypothesis that trace elements are driven by diet. An increase in trace element uptake in the summer season also favors the hypothesis that increased dust input forces higher trace element concentrations in the environment in summer, which are then incorporated into the enamel with a slight (2–3 month) lag in time ([89] [91]). The time lag can in this case be explained in part by longer residence times of trace elements in the environment, as part of the trace element input will be taken up by the horse indirectly through the vegetation ([87] [131] [133]). The data provided here seems to suggest that both dust input into the environment and changes in diet over the year are sources of trace element variations in the horse specimen. Further research into the pathways of trace element uptake and incorporation into bioapatite would be needed to confirm the most important mechanism explaining trace element seasonality in horse molars. The best way to conduct such a study for large mammals would be to allow growth of the animals under fully controlled circumstances and to measure stable isotope ratios and trace element concentrations of all sources of food and water. Such a study would be time consuming and potentially ethically complicated.

### 5.6 Enamel thickness and depth integration

The offset of K/Ca and Zn/Ca values of P^2^ and Fe/Ca of P^3^ shown in Table 2 could indicate that the rate of incorporation of trace elements is variable with tooth position. However, this is unlikely because the offset is not observed equally in all elements and is not consistent within one tooth. Another reason for the difference is posed by [83] who found cross sectional gradients in trace element concentrations through mammal teeth. If such gradients exist in horse teeth, a variation in the thickness of the enamel between different teeth is expected to significantly change the trace element abundance measured with μXRF. Since the attenuation depth of X-Ray radiation increases with atomic weight ([134] [135]), lighter elements are measured more superficially than heavier elements. A change in the inward gradient of trace element concentrations resulting from a thinner (more condensed) enamel layer or the removal of the outer (more trace element-rich) part of the enamel will therefore result in a larger change in the measurements of lighter elements compared to heavier elements. The effect of enamel thinning is dampened in measurements of heavier elements because they are more depth-averaged. The fact that trace element ratios in P^2^ are higher than in other teeth while Fe/Ca in P^3^ is lower (Table 2) may therefore indicate that the enamel layer measured in P^2^ was thicker than average and that of P^3^ was thinner and more condensed than in the other teeth. Table 2 reveals that the other trace element records of P^2^ also have elevated concentrations, supporting the hypothesis of a thicker enamel and a relatively bigger contribution of the trace element-enriched outer enamel layer. The preparation of the teeth for XRF scanning by cleaning off the most contaminated outer layer of the tooth could also result in differential loss of outer enamel between different teeth. However, since the amount of enamel removed by this cleaning procedure is very limited (<0.1 mm) it is not likely to cause the large offset in trace element concentration reported in Fig 5 and Table 2. For future studies it is recommended that such pre-cleaning of teeth for XRF, or any other surface-based analysis, is done by air-abrasion to allow even more control on the amount of enamel to be removed. Another possibility is that analysis are executed on enamel cross sections, although this does compromise the non-destructive character of the XRF measurement and might as such not be a favorable procedure on precious samples. Cross-sectional transects through modern horse teeth could also reveal whether an inward gradient in trace elemental abundance is present in horse enamel, supporting this hypothesis.

Because the enamel mineralization front in mammal molars is not perpendicular to the growth direction of the tooth, variations in depth integration between elements also influence time averaging in the sample volume ([136]). Work by [21] elegantly shows how samples taken perpendicular to the tooth surface combine enamel that mineralized over a larger period of time. Based on their work and information about the slope of the mineralization front in horse molars (5 to 10 degrees; [34]) and their growth rate (3–4 mm/yr; [34]), measurements of heavier elements like Sr and Zn with attenuation lengths of 100s of microns will average about 2–3 months of enamel formation while lighter elements like Mg and Si with attenuation lengths of a few microns will average only several days of enamel formation. As a result, seasonality curves for heavier elements are more smoothed than those of lighter elements. Besides, shallower samples (lighter elements) also sample comparatively younger enamel than deeper samples (see also modelling and discussion in [21]). The difference in average age is around 1–2 months and may explain a lag of the seasonality of heavier elements like Zn and Sr with respect to lighter elements like Fe and S. Other, less penetrative, techniques for determining trace element abundance can be attempted to show whether depth-integration causes the offsets observed between teeth.

### 5.7 Broader implications for further research

The discovery of seasonally fluctuating trace element concentrations in mammal teeth opens up the possibility for the development of new paleo-dietary and/or paleo-environmental proxies. The preliminary results presented in this paper clearly show that the measurement of trace element profiles in mammal tooth enamel with the new μXRF line-scanning technique is feasible within a short timeframe without physical alteration of the samples. While further investigation is required to confidently interpret seasonal trace element profiles in mammal enamel, the current study clearly shows that there are seasonal patterns in several common trace elements that will be of interest for future archaeological and palaeontological research. Once established, trace element proxies in bioapatite could be used in combination with or as a replacement of stable isotope analyses as they have often been used in studies of seasonally resolved carbonate records (e.g. [24] [25] [26] [27] [137] and references therein). Trace element profiles in tooth enamel could then play a role in a wide range of paleo-dietary and paleo-environmental studies aiming to solve questions of past human and animal migration, paleo-diet and seasonal variations in paleo-environment.

## 6. Conclusions

μXRF scanning on the cleaned surface of mammal molars and premolars yields repeatable values for trace elements heavier than Mg and with a concentration of 1 ppm or higher. Lighter elements and lower concentrations cause reduced repeatability of the quantification of μXRF results. Results of stable oxygen isotope ratios indicate that seasonal changes in temperature and consequently in the oxygen isotope ratio of precipitation are faithfully recorded in this modern horse molars and premolars. This relationship can be used together with eruption patterns to construct an age model allowing the creation of a composite multi-year record from multiple teeth of the same individual. Such a composite record shows that records of Sr/Ca, Zn/Ca, K/Ca, Fe/Ca and S/Ca in all teeth reflect the same seasonal pattern. This seasonal fluctuation in elemental abundance records seems to be in phase for all elements and shows a slight phase lag of approximately 2–3 months with respect to oxygen isotopes and temperature seasonality. Carbon isotopes from the same teeth exhibit seasonality in approximate antiphase with respect to oxygen and trace elements and are related to diet.

Two possible mechanisms for the seasonality in trace elements are proposed. On the one hand, seasonal fluctuations in the composition of the horse’s diet can influence the amount of trace elements taken up by the animal on a seasonal scale. One the other hand, a seasonal fluctuation in the amount of available dust and the concentration of trace elements in dust particles could be incorporated into the animal either through direct dust intake, as suggested by [83] or indirectly through leaching of trace elements from dust into the local environment and incorporation into the animal’s diet (e.g. through consumed grass). This external forcing of trace element composition in mammal tooth enamel implies that trace element ratios may be used as proxies for seasonal changes in paleo-environment and paleo-diet. This discovery potentially opens up a whole set of new proxies in bioapatite that are relatively easy and rapid to measure and yield information that is complementary to conventional isotope proxies. Further research is recommended to determine the dominant pathway of trace element incorporation into mammal tooth enamel and to determine whether the same seasonal patterns in trace element concentrations can be found in archaeological or fossil tooth samples.

## Supporting Information

S1 File Mass spectrometry and μXRF results of P^2^. (XLSX)

S2 File Mass spectrometry and μXRF results of P^3^. (XLSX)

S3 File Mass spectrometry and μXRF results of P^4^. (XLSX)

S4 File Mass spectrometry and μXRF results of M^1^. (XLSX)

S5 File Mass spectrometry and μXRF results of M^2^. (XLSX)

S6 File Mass spectrometry and μXRF results of M^3^. (XLSX)

S7 File Horse drinking water quality restrictions in Flanders. (PDF)

S8 File Certified values of BAS-CCB01 reference material. (PDF)

S9 File Graph of attenuation lengths of X-Rays at energies that are representative of analyzed elements and table of attenuation lengths for each element. (PDF)
