# Supplementary material for: Seasonal Cyclicity in Trace Elements and Stable Isotopes of Modern Horse Enamel
Source: PLoS One. 2016 Nov 22;11(11):e0166678. doi: 10.1371/journal.pone.0166678 (PMC5119779; doi:10.1371/journal.pone.0166678)
Supplement: S7 File — (PDF) [file pone.0166678.s007.pdf]

Normen wateronderzoek: ZOOGDIEREN & IKM

Chemisch onderzoek	Varkens	Paarden	Herkauwers	IKM drinkwater	IKM reinigingswater
Fysisch aspect	Helder; kleur - en geurloos	Helder; kleur - en geurloos	Helder; kleur - en geurloos	/	/
pH	4 - 9	6,5 - 8	5,5 - 8,5	/	/
Geleidbaarheid	2100 µS/cm	2100 µS/cm	2100 µS/cm	/	/
Totale hardheid	Max. 20°D	Max. 20°D	Max. 20°D	/	/
Fluoride	≤ 1,5 mg/l	≤ 2,0 mg/l	≤ 2,0 mg/l	/	/
Chloride	≤ 250 mg/l	≤ 250 mg/l	≤ 250 mg/l	/	/
Nitriet	≤ 0,5 mg/l	≤ 0,5 mg/l	≤ 1,0 mg/l	< 1,0 mg/l	< 0,5 mg/l
Nitraat	≤ 200 mg/l	≤ 200 mg/l	≤ 200 mg/l	< 200 mg/l	< 50 mg/l
Fosfaat	≤ 5,0 mg/l	≤ 5,0 mg/l	≤ 2,0 mg/l	/	/
Sulfaat	≤ 250 mg/l	≤ 250 mg/l	≤ 250 mg/l	/	/
Sulfide	afwezig	afwezig	afwezig	/	/
Ammonium	≤ 2,0 mg/l	≤ 2,0 mg/l	≤ 10 mg/l	/	/
Totaal ijzer	≤ 2,5 mg/l	≤ 2,5 mg/l	≤ 2,5 mg/l	/	/
Mangaan	≤ 1,0 mg/l	≤ 1,0 mg/l	≤ 1,0 mg/l	/	/
Magnesium	≤ 50 mg/l	≤ 50 mg/l	≤ 50 mg/l	/	/
Calcium	≤ 270 mg/l	≤ 270 mg/l	≤ 270 mg/l	/	/
Natrium	≤ 400 mg/l	≤ 400 mg/l	≤ 400 mg/l	/	/
Zoutgehalte	≤ 3000 mg/l	≤ 3000 mg/l	≤ 3000 mg/l	/	/

Bacteriologisch onderzoek	Varkens	Paarden	Herkauwers	IKM drinkwater	IKM reinigingswater
Tot kiemgetal 22°C	< 100.000 kve / ml	< 100.000 kve / ml	< 100.000 kve / ml	/	/
Tot kiemgetal 37°C	< 100.000 kve / ml	< 100.000 kve / ml	< 100.000 kve / ml	< 100.000 kve / ml	< 100 kve / ml
Coliformen	< 100 kve / ml	< 100 kve / ml	< 100 kve / ml	< 100 kve / ml	< 10 kve / 100 ml
E. coli	< 100 kve / ml	< 100 kve / ml	< 100 kve / ml	/	< 1 kve/ 100 ml
Intestinale enterococcen	< 1 kve / 100 ml	< 1 kve / 100 ml	< 1 kve / 100 ml	/	/
Sulfiet red. Clostridia	< 1 kve / 20 ml	< 1 kve / 20 ml	< 1 kve / 20 ml	/	/
C. perfringens	< 1 kve / 100 ml	< 1 kve / 100 ml	< 1 kve / 100 ml	/	/
Schimmels/gisten	< 10.000 kve/ml	< 10.000 kve/ml	< 10.000 kve/ml	/	/
Salmonella sp.	afwezig	afwezig	afwezig	/	/
