# Supplementary material for: Seasonal Cyclicity in Trace Elements and Stable Isotopes of Modern Horse Enamel
Source: PLoS One. 2016 Nov 22;11(11):e0166678. doi: 10.1371/journal.pone.0166678 (PMC5119779; doi:10.1371/journal.pone.0166678)
Supplement: S8 File — (PDF) [file pone.0166678.s008.pdf]

LUCIDEON
insight creating advantage

DATA SHEET· CCB1 – CALCINED BONE CHEMICAL ANALYSIS

February 2012

The main chemical analysis by Lucideon, a Laboratory Accredited to EN ISO/IEC 17025 by UKAS and using the XRF Standard BS EN ISO 12677: 2011. Fluorine was by pyrohydrolysis and Ion Selective Electrode. Sulphur was by Leco. Results are on the dried 110°C basis. Each sub-sample was analysed in duplicate.

Constituent	Formula	Mean	SD	Sub-samples
		Wt %		
Silicon Dioxide	SiO_2	1.28	0.04	12
Titanium Dioxide	TiO_2	<0.01		12
Alumina	Al_2O_3	0.05	0.01	12
Iron (III) Oxide	Fe_2O_3	0.04	0.01	12
Calcium Oxide	CaO	53.4	0.3	12
Magnesium Oxide	MgO	1.14	0.02	12
Potassium Oxide	K_2O	0.011	0.001	12
Sodium Oxide	Na_2O	0.52	0.03	12
Phosphorus Pentoxide	P_2O_5	40.5	0.3	12
Barium Oxide	BaO	0.031	0.004	12
Strontium Oxide	SrO	0.049	0.004	12
Sulphur Trioxide	SO_3	0.114	0.008	3
Fluorine	F	0.13	0.01	3
(Moisture)		(0.12)	0.04	12
LoI 110°C to 1025°C		2.60	0.07	12
Total		99.92		

SO_3 by XRF was 0.113%. XRD found 0.5% Quartz. Hydroxyapatite was the major constituent and estimated as 99.5% (by difference).
