# Supplementary material for: Seasonal Cyclicity in Trace Elements and Stable Isotopes of Modern Horse Enamel
Source: PLoS One. 2016 Nov 22;11(11):e0166678. doi: 10.1371/journal.pone.0166678 (PMC5119779; doi:10.1371/journal.pone.0166678)
Supplement: S9 File — (PDF) [file pone.0166678.s009.pdf]

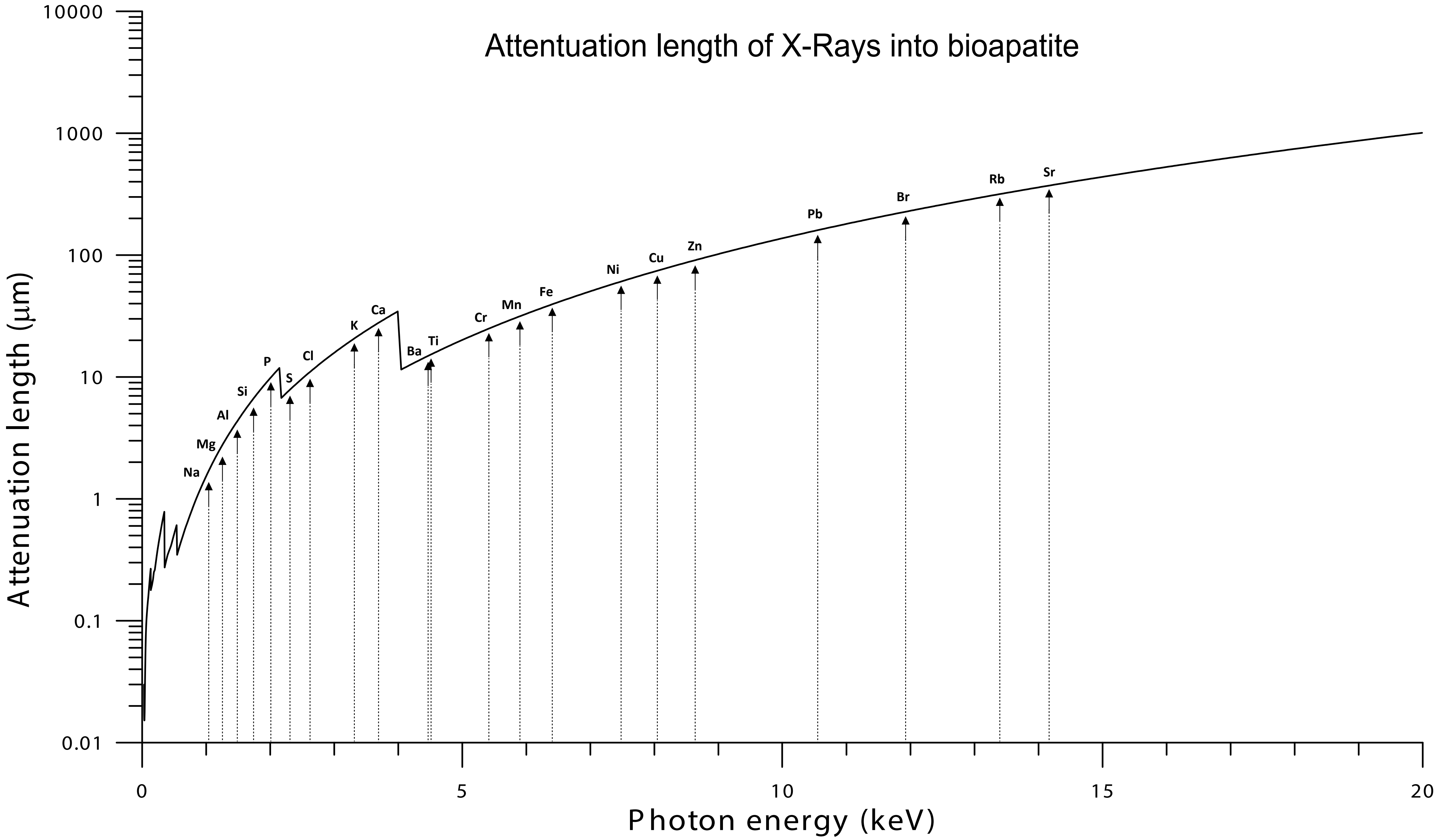

element	Na	Mg	Al	Si	P	S	Cl	K	Ca	Ba	Ti	Cr	Mn	Fe	Ni	Cu	Zn	Pb	Br	Rb	Sr
keV	1.04	1.25	1.49	1.74	2.01	2.31	2.62	3.31	3.69	4.47	4.51	5.42	5.90	6.41	7.48	8.05	8.64	10.55	11.92	13.40	14.17
L_{att}(μm)	1.70	2.77	4.34	6.67	9.92	7.88	11.01	20.70	27.82	14.90	15.31	24.95	31.51	39.48	60.71	74.45	90.85	160.10	226.94	316.74	371.95
